# Relative Hyperlactatemia in the Emergency Department

**DOI:** 10.3389/fmed.2020.00561

**Published:** 2020-09-22

**Authors:** Ralphe Bou Chebl, Sarah Jamali, Nancy Mikati, Reem Al Assaad, Karim Abdel Daem, Nadim Kattouf, Rawan Safa, Maha Makki, Hani Tamim, Gilbert Abou Dagher

**Affiliations:** ^1^Department of Emergency Medicine, American University of Beirut Medical Center, Beirut, Lebanon; ^2^Department of Internal Medicine, Clinical Research Institute, American University of Beirut, Beirut, Lebanon

**Keywords:** lactate, mortality, emergency & critical care, morbidity, sepsis

## Abstract

**Objective:** The clinical interpretation of lactate ≤ 2.00 mmol/L in emergency department (ED) patients is not well-characterized. This study aims to determine the optimal cutoff value for lactate within the reference range that predicts in-hospital mortality among ED patients.

**Methods:** This was a retrospective study of adult patients presenting to a tertiary ED with an initial serum lactate level of <2.00 mmol/L. The primary outcome was in-hospital mortality. Youden's index was utilized to determine the optimal threshold that predicts mortality. Patients above the threshold were labeled as having relative hyperlactatemia.

**Results:** During the study period, 1,638 patients were included. The mean age was 66.9 ± 18.6 years, 47.1% of the population were female, and the most prevalent comorbidity was hypertension (56.7%). The mean lactate level at presentation was 1.5 ± 0.3 mmol/L. In-hospital mortality was 3.8% in the overall population, and 16.2% were admitted to the ICU. A lactate level of 1.33 mmol/L was found to be the optimal cutoff that best discriminates between survivors and non-survivors. Relative hyperlactatemia was an independent predictor of in-hospital mortality (OR 1.78 C1.18–4.03; p = 0.02). Finally, relative hyperlactatemia was associated with increased mortality in patients without hypertension (4.7 vs. 1.1%; *p* = 0.008), as well as patients without diabetes or COPD.

**Conclusion:** The optimal cutoff of initial serum lactate that discriminates between survivors and non-survivors in the ED is 1.33 mmol/L. Relative hyperlactatemia is associated with increased mortality in emergency department patients, and this interaction seems to be more important in healthy patients.

## Learning Points

- This study aims to determine the optimal cutoff value for lactate within the reference range that predicts in-hospital mortality among emergency (ED) patients with initial serum lactate levels within the reference range. One thousand six hundred thirty-eight patients were included. The mean age was 66.9 ± 18.6 years; 47.1% of the population were female.
- Relative hyperlactatemia was an independent predictor of in-hospital mortality (OR 1.78 CI 1.18–4.03; *p* = 0.02) in patients presenting to the emergency department.
- The lactate optimal cutoff of 1.33 mmol/L was found to be the optimal cutoff that best discriminates between survivors and non-survivors.
- Relative hyperlactatemia is associated with increased mortality in emergency department patients, and this interaction seems to be more important in healthy patients.

## Introduction

### Background

The breakdown of pyruvate via the enzyme lactate dehydrogenase leads to the formation of lactate. Healthy individuals produce basal lactate levels of 1.0 ± 0.5 mmol/L (1, 2). Normal lactate levels in the blood usually refer to levels <2 mmol/L (3, 4). Current theories relate hyperlactatemia to decreased oxygen delivery and tissue malperfusion, or to impaired oxygen utilization and adrenergic stress, and both paradigm mechanisms may be compounded by impaired elimination (5–10). There have been significant advances in our understanding of the physiology of lactate, and it has since become a mainstay biomarker, heavily integrated into clinical decision making of septic patients in the emergency department (11, 12). Furthermore, hyperlactatemia (>2.00 mmol/L) has been associated with poor outcomes and independently predicts mortality in diverse patient populations presenting to the emergency department (ED) (13). In its most recent guidelines, the Surviving Sepsis Campaign recommends using lactate levels ≥4 mmol/L to initiate IV fluid resuscitation and recommends remeasuring lactate levels if they are >2 mmol/L to monitor the response to resuscitation (8).

### Importance

Lactate levels within the reference range ( ≤ 2 mmol/L) have a less clear clinical interpretation and may result in less attention given to these patients in the ED. Furthermore, there is a considerable group of patients who present with shock and have elevated in-hospital mortality rates despite having lactate levels within the normal range (14). It has been proposed that this subgroup of patients with septic shock possesses distinctive clinical and physiological profiles, and may have unique treatment parameter considerations (15–17). Moreover, there is emerging evidence that suggests that relative hyperlactatemia (i.e., lactate above an identified threshold) has a more appropriate consideration in certain subgroups of patients, such as those with sepsis (18), septic shock (14, 19), or cancer (20). The clinical interpretation of lactate ≤ 2.00 mmol/L in ED patients is not well-characterized. This allows us to question what the best prognostic cutoff value is for lactate. One study proposed that a cutoff of 1.35 mmol/L best discriminates between survivors and non-survivors in the intensive care unit (ICU) (21). Nonetheless, there is a paucity of data on this issue, particularly in the ED.

### Goals of This Investigation

This study aims to evaluate the optimal cutoff threshold for lactate that distinguishes between survivors and non-survivors and predicts in-hospital mortality among patients presenting to the ED with initial serum lactate levels within the reference range (0.01–2.00 mmol/L).

## Methods

### Study Design and Setting

This was a retrospective cohort study of adult patients presenting to the academic ED of a tertiary care center between the dates of January 1, 2017 and June 30, 2019. All patients aged 18 years of age or older who presented to the ED and had a serum lactate level drawn had their charts queried. All patients who had an initial serum lactate level drawn and within the reference range (0.01–2.00 mmol/L) were included in the study. Exclusion criteria consisted of patients with serum lactate >2.00 mmol/L, patients who were pregnant, patients who presented with cardiac arrest, and patients who had been admitted <10 days prior to presentation. The data collection protocol was standardized, and information was extracted from the patient's electronic medical records and anonymized. The variables collected included patient demographics and characteristics, vital signs, and initial laboratory tests upon presentation to the ED, diagnosis, presence of sepsis on admission, interventions performed (renal replacement therapy, mechanical ventilation, vasopressor use, steroid use, antibiotic administration, intravenous fluid administration), disposition, length of stay, readmission rates, in-hospital mortality, and 30-day mortality rates. In this study, sepsis was defined as the presence of an infection with signs of organ dysfunction, as represented by the Sequential (Sepsis-related) Organ Failure Assessment (SOFA) score of 2 points or greater according to the Third International Consensus Definitions for Sepsis and Septic Shock (Sepsis-3) guidelines (22). Patients who did not meet this definition were labeled as having an infection. The study was approved by the hospital's Institutional Review Board (IRB; BIO-2018-0453). Patients or the public were not involved in the design, conduct, reporting, or dissemination plans of our research.

### Outcomes

The primary outcome was in-hospital mortality. The secondary outcomes included mechanical ventilation, vasopressor use, steroid use, intravenous fluid administration, and lengths of stay (ED, ICU, and total).

### Statistical Analysis

Statistical analysis was conducted with IBM SPSS Statistics for Windows, version 24 (IBM Corp., Armonk, N.Y., USA). Continuous variables are presented as mean ± standard deviation, and categorical variables are presented as frequency with valid percent. Patients were stratified into survivors and non-survivors. A Youden's index was used to determine the optimal threshold that predicts in-hospital mortality, and patients above the threshold were reclassified as having relative hyperlactatemia. A multivariate logistic regression was performed to determine the association of relative hyperlactatemia and in-hospital mortality. All variables with statistical and clinical significance were included in the analysis. The variables included were age, gender, comorbidities (hypertension, diabetes, dyslipidemia, chronic obstructive pulmonary disease, heart failure, immunocompromised), diagnosis category, sepsis, lymphocyte count, and WBC count. We looked at the in-hospital mortality among patients with and without relative hyperlactatemia, stratified by selected subgroups. These subgroups included the following: male vs. female patients, age younger than 50 years vs. age older or equal to 50 years, diabetes vs. no diabetes, hypertension vs. no hypertension, dyslipidemia vs. no dyslipidemia, coronary artery disease vs. no coronary artery disease, COPD vs. no COPD, congestive heart failure vs. no congestive heart failure, sepsis vs. no sepsis, and vasopressor vs. no vasopressor use.

## Results

**Figure 1:**
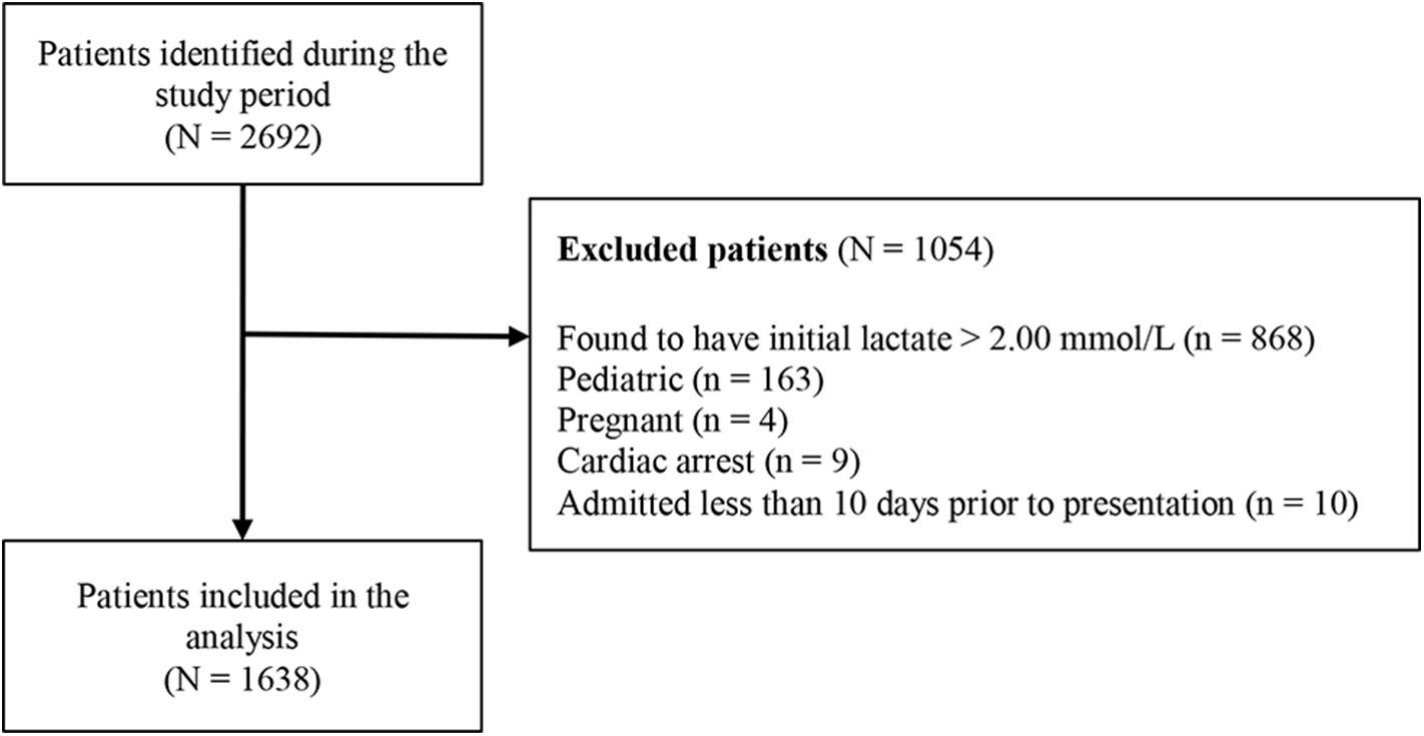
Flow diagram of patient selection.

During the study period, a total of 2,692 patients were identified with lactate levels within the reference range (0.01–2.00 mmol/L). Of these, 1,054 patients were excluded, with the exclusion reasons shown in Figure 1. A total of 1,638 patients were included in the study, and their characteristics are summarized in Table 1.

**Table 1:** Baseline characteristics, vital signs, laboratory values, and outcomes for all patients with lactate ≤ 2.00 mmol/L.

**Variable**	**Overall *N* = 1,627**
	Mean ± SD
Age (years)	66.89 ± 18.61
	*n*, %
Sex (female)	767 (47.1)
**COMORBIDITIES**	
Hypertension	921 (56.7)
Dyslipidemia	504 (31.1)
Coronary artery disease	409 (25.2)
Diabetes mellitus	473 (29.1)
CKD	229 (14.1)
ESRD	60 (3.7)
Hepatic dysfunction	16 (1.0)
COPD	167 (10.3)
Malignancy	360 (22.1)
Congestive heart failure	215 (13.2)
**DIAGNOSIS CATEGORY**	
Respiratory	188 (11.6)
Cardiovascular	95 (5.8)
Neurologic	53 (3.3)
Trauma	60 (3.7)
Infection	714 (43.9)
Gastrointestinal	294 (18.1)
Other medical illness	222 (13.6)
Sepsis	517 (31.8)
	Mean ± SD
**VITAL SIGNS AT PRESENTATION**	
SBP (mm Hg)	127.48 ± 25.51
DBP (mm Hg)	69.04 ± 15.26
HR (per minute)	92.81 ± 21.57
Oxygen saturation (%)	96.26 ± 4.83
Temperature (°C)	37.33 ± 0.92
**LABORATORY RESULTS**	
Lactate at presentation (mmol/L)	1.47 ± 0.25
Glucose (mg/dl)	129.54 ± 64.20
Creatinine (mg/dl)	1.47 ± 1.63
WBC (/cu.mm)	10,903.76 ± 6,699.65
pH (arterial)	7.38 ± 0.09
INR	1.48 ± 0.88
	*n*, %
**OUTCOMES**	
Mechanical ventilation	51 (3.1)
Vasopressor use	69 (4.2)
Steroid use	188 (11.6)
ICU admission	264 (16.2)
30-Day readmission rate	329 (22.1)
In-hospital mortality	60 (3.8)
	Mean ± SD
IV fluids in first 6 h	1.255 ± 1.16
IV fluids in first 24 h	1.94 ± 1.74
Length of stay (h)	123.85 ± 261.32
ED	9.22 ± 13.37
ICU	31.96 ± 173.50
GPU	82.67 ± 186.17

*INR, international normalized ratio; BUN, blood urea nitrogen; PaO_2_, partial pressure of oxygen; FiO_2_, fraction of inspired oxygen; ICU, intensive care unit; IV, intravenous; ED, emergency department; GPU, general practitioner unit*.

### Overall Patient Characteristics

The mean age was ~66.9 ± 18.6 years. Of the population, 47.1% were female, and the most prevalent comorbidity was hypertension (56.7%). Of the patients, 43.9% had an infection, and 31.8% of all the patients had a diagnosis of sepsis. The mean lactate level at presentation was 1.5 ± 0.3 mmol/L. During their hospital stay, 3.8% of the patients died, 4.2% received vasopressors, 3.1% were mechanically ventilated, and 16.2% were admitted to the ICU. The mean length of stay in the ED was 9.2 ± 13.4 h.

### ROC Curve

**Figure 2:**
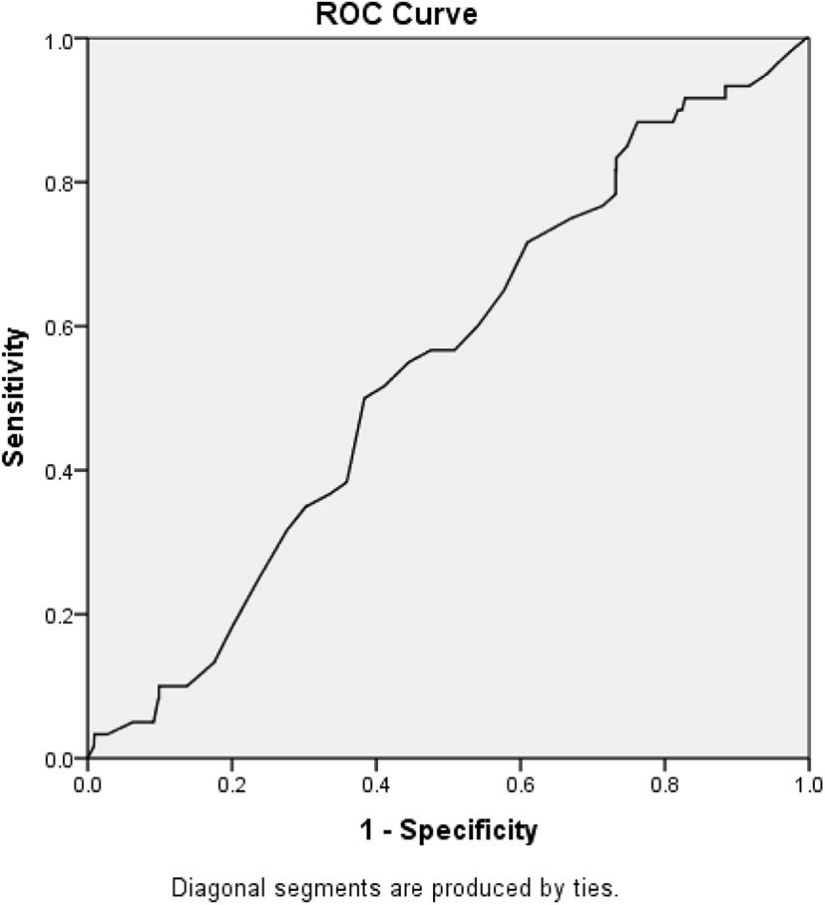
Receiver operating curve and area under the curve. The optimal cutoff that differentiates between survivors and non-survivors was found to be 1.33, and the area under the curve for that value was 0.545 (95% CI 0.477–0.614).

Figure 2 demonstrates the receiver operating curve for lactate upon presentation and in-hospital mortality. The optimal cutoff that differentiates between survivors and non-survivors was found to be 1.33, and the area under the curve for that value was 0.545 (95% CI 0.477–0.614).

### Laboratory and Vital Signs

Youden's index was used to find the threshold that best discriminates between survivors and non-survivors, and it was found to be 1.33 mmol/L. Table 2 summarizes the characteristics of patients with initial lactate levels below and above 1.33 mmol/L. Patients with relative hyperlactatemia (equal to or above 1.33 mmol/L) were older (68.8 ± 17.8 years vs. 61.4 ± 19.8, *p* < 0.001), had more comorbidities, higher heart rates and temperatures, lower oxygen saturations, higher glucose, and WBC count than patients with lactate levels below 1.33 mmol/L.

**Table 2:** Patient characteristics, vital signs, and laboratory values upon ED presentation for patients with lactate <1.33 mmol/L and for patients with lactate ≥1.33 mmol/L.

**Variable**	**Lactate <1.33 *n* = 415**	**Lactate ≥ 1.33 *n* = 1,212**	***p***
	Mean ± SD		
Age (years)	61.36 ± 19.78	68.79 ± 17.82	<0.001
	*n*, %		
Sex (female)	215 (51.8)	552 (45.4)	0.027
**COMORBIDITIES**			
Hypertension	206 (49.6)	715 (59.1)	0.001
Dyslipidemia	109 (26.3)	395 (32.7)	0.015
Coronary artery disease	83 (20.0)	326 (27.0)	0.005
Diabetes mellitus	87 (21.0)	386 (31.9)	<0.001
Chronic kidney disease	52 (12.6)	177 (14.6)	0.307
ESRD	13 (3.1)	47 (3.9)	0.490
Hepatic dysfunction	4 (1.0)	12 (1.0)	1.000
COPD	29 (7.0)	138 (11.4)	0.011
Malignancy	88 (21.2)	272 (22.5)	0.584
Congestive heart failure	23 (5.6)	192 (15.9)	<0.001
**DIAGNOSIS CATEGORY**			
Respiratory	35 (8.4)	153 (12.6)	<0.001
Cardiovascular	14 (3.4)	81 (6.7)	
Neurologic	20 (4.8)	33 (2.7)	
Operative trauma	0 (0.0)	8 (0.7)	
Non-operative trauma	22 (5.3)	30 (2.5)	
Infection	154 (37.1)	560 (46.2)	
Gastrointestinal	97 (23.4)	197 (16.3)	
Other medical illness	73 (17.6)	149 (12.3)	
Sepsis APACHE II	105 (25.3) c19.6 ± 6.0	412 (34)c 22.3 ± 5.4	<0.001 <0.001
	Mean ± SD		
**VITAL SIGNS AT PRESENTATION**			
SBP (mm Hg)	125.52 ± 23.44	128.15 ± 26.15	0.057
DBP (mm Hg)	68.79 ± 14.17	69.13 ± 15.62	0.678
HR (per minute)	90.99 ± 20.28	93.43 ± 21.97	0.039
Oxygen saturation (%)	97.38 ± 3.70	95.88 ± 5.11	<0.001
Temperature (°C)	37.24 ± 0.81	37.35 ± 0.96	0.024
**LABORATORY RESULTS**			
Lactate at presentation mmol/L	1.11 ± 0.15	1.59 ± 1.34	<0.001
Glucose mg/dl	117.76 ± 65.00	134.03 ± 63.36	<0.001
Creatinine mg/dl	1.42 ± 1.97	1.49 ± 1.49	0.460
WBC/cu.mm	9,861.23 ± 5,961.54	11,259.01 ± 6,899.63	<0.001
pH (Arterial)	7.36 ± 0.10	7.38 ± 0.09	0.073
INR	1.31 ± 0.61	1.52 ± 0.93	0.001

*INR, international normalized ratio; BUN, blood urea nitrogen; PaO_2_, partial pressure of oxygen; FiO_2_, fraction of inspired oxygen*.

Patients with relative hyperlactatemia were also more likely to have a diagnosis of sepsis (56.2 vs. 34.1% *p* < 0.001), and they had a higher APACHE II score (19.6 ± 6.0 vs. 22.3 ± 5.4, *p* < 0.001). Furthermore, they were more likely to receive vasopressors (5.0 vs. 2.2%, *p* = 0.015), steroids (13.0 vs. 7.5%, *p* < 0.001), and more IV fluids in the first 24 h (2.1 ± 1.2, *p* < 0.001) compared to patients with lactate levels below 1.33 mmol/L. In-hospital mortality was higher in patients with relative hyperlactatemia (4.4 vs. 1.9%, *p* = 0.029) compared to patients with lactate levels below 1.33 mmol/L. The outcomes are summarized in Table 3.

**Table 3:** Outcomes for patients with lactate <1.33 mmol/L and for patients with lactate ≥1.33 mmol/L.

**Variable**	**Lactate <1.33 *n* = 415**	**Lactate ≥ 1.33 *n* = 1,212**	***p***
	*n*, %		
Mechanical ventilation	11 (2.7)	40 (3.3)	0.514
Vasopressor use	9 (2.2)	60 (5.0)	0.015
Steroid use	31 (7.5)	157 (13.0)	0.003
Antibiotic use	179 (43.2)	848 (70.1)	<0.001
ICU admission	35 (23.2)	229 (24.7)	0.681
30-Day readmission rate	86 (23.8)	243 (21.6)	0.376
In-hospital mortality	7 (1.9)	53 (4.4)	0.029
	Mean ± SD		
IV fluids in first 6 h	1.21 ± 1.08	1.27 ± 1.19	0.308
IV fluids in first 24 h	1.58 ± 1.52	2.07 ± 1.79	<0.001
	Mean ± SD		
Length of stay (h)	73.62 ± 190.96	141.05 ± 279.40	<0.001
ED	7.89 ± 12.18	9.67 ± 13.73	0.013
ICU	14.62 ± 110.22	37.90 ± 190.07	0.003
GPU	51.11 ± 134.21	93.48 ± 190.96	<0.001

*ICU, intensive care unit; IV, intravenous; ED, emergency department; GPU, general practitioner unit*.

### Multivariate Logistic Regression

After adjusting for the multiple confounding variables such as age, gender, laboratory results, and comorbidities, we found that patients with relative hyperlactatemia had 1.78 greater odds of in-hospital mortality (95% CI 1.18–4.03; *p* = 0.02) than patients without (Table 4).

**Table 4:** Multivariate logistic regression adjusting for multiple characteristics and outcomes with the primary outcome as in-hospital mortality and the primary exposure as relative hyperlactatemia.

	**In-hospital mortality**			
	**OR**	**95% C.I**.		***p***
		**Lower**	**Upper**	
Lactate > 1.33	1.78	1.18	4.03	0.02
Dyslipidemia	0.360	0.171	0.759	0.007
Congestive heart failure	6.483	3.462	12.141	<0.001
Immunocompromised	2.544	1.179	5.492	0.017

*Stepwise: age, gender (reference: male), hypertension, diabetes, dyslipidemia, COPD, heart failure, immunocompromised; diagnostic category: respiratory, cardiovascular, neurologic, trauma, sepsis, gastrointestinal, lymphocyte count, WBC count*.

### Subgroup Analysis

The association between relative hyperlactatemia and mortality in various subgroups is demonstrated in Table 5. Relative hyperlactatemia was associated with increased hospital mortality consistently across the different subgroups; however, the difference was only statistically significant in patients without hypertension (4.7 vs. 1.1%; *p* = 0.008), patients without diabetes (4.2 vs. 1.0%; *p* = 0.01), patients without dyslipidemia (5.4 vs. 1.5%; *p* = 0.008), and patients without COPD (4.3 vs. 1.8%; *p* = 0.04).

**Table 5:** In-hospital mortality among patients with lactate <1.33 mmol/L and patients with lactate >1.33 mmol/L stratified by different patient subgroups.

**Patient subgroup**		**Lactate <1.33 mmol/L**	**Lactate ≥ 1.33 mmol/L**	***p*-value**
Age	<50	1 (1.0)	7 (3.6)	0.27
	≥50	6 (2.3)	46 (4.6)	0.09
Sex	Male	4 (2.2)	29 (4.5)	0.18
	Female	3 (1.6)	24 (4.4)	0.08
Diabetes	Yes	4 (5.3)	19 (5.0)	0.92
	No	3 (1.0)	34 (4.2)	0.01
HTN	Yes	5 (2.7)	30 (4.3)	0.33
	No	2 (1.1)	23 (4.7)	0.03
Dyslipidemia	Yes	3 (3.1)	9 (2.3)	0.65
	No	4 (1.5)	43 (5.4)	0.008
Immunocompromised	Yes	0 (0.0)	11 (7.6)	0.45
	No	7 (2.0)	41 (4.0)	0.08
CAD	Yes	2 (2.7)	20 (6.3)	0.23
	No	5 (1.7)	33 (3.8)	0.09
COPD	Yes	1 (3.4)	8 (5.8)	0.61
	No	6 (1.8)	45 (4.3)	0.04
CHF	Yes	0 (0.0)	25 (13.2)	0.07
	No	7 (2.1)	28 (2.8)	0.46
Sepsis	Yes	3 (2.4)	34 (5.9)	0.11
	No	3 (1.4)	14 (3.2)	0.17
Vasopressors	Yes	1 (11.1)	9 (15.3)	0.74
	No	6 (1.7)	44 (3.9)	0.046

## Discussion

The results of this study have shown that lactate levels of 1.33 mmol/L were found to have the optimal threshold to discriminate between survivors and non-survivors. Furthermore, patients with relative hyperlactatemia (1.33–2.00 mmol/L) had 1.78 times greater odds of dying than patients with lactate <1.33 mmol/L. The overall hospital mortality in our population was 3.8%, with the relative hyperlactatemia subgroup having a higher mortality rate of 4.4% in patients with initial lactate ≥1.33 mmol/L, compared to 1.9% in patients with initial lactate <1.33 mmol/L. Similar results were demonstrated in a study by Rishu et al. who looked at a discriminatory level of lactate in an intensive care unit and found that a cutoff value of 1.35 mmol/L adequately discriminated between survivors and non-survivors (21). In addition, they also found that mild hyperlactatemia was an independent predictor of hospital mortality (OR 1.60; 95% CI 1.29–1.98). Furthermore, it is also interesting that among the subgroup analyses, relative hyperlactatemia was significantly associated with a higher mortality in patients without comorbidities, when compared to lactate levels <1.33. In the literature, lactate's prognostic role is more important in older patients with comorbidities (13, 23). Our finding is very interesting and important as it illustrates the important prognostic value of relative hyperlactatemia in healthy individuals vs. patients with comorbidities. A possible explanation could be that patients with comorbidities are metabolically sicker at baseline due to the burden of their chronic illnesses and tend to raise their lactate more easily in the setting of an acute illness, whereas healthier patients might not have high lactates or have lactates of 0.2 mmol/L, and the mere fact that they raise lactate levels to >1.3 mmol/L should raise an alarm with the treating physicians.

Defining hyperlactatemia as a serum lactate level ≥2.00 mmol/L insinuates that lactate values between 1.00 and 2.00 mmol/L can be interpreted as normal (16, 22). Historically, authors have used the cutoff of 1.3 mmol/L to define hyperlactatemia (2, 24). Over the years, the reference ranges for hyperlactatemia have varied from lactate >1.5 mmol/L to lactate >2.5 mmol/L (15, 19, 25). Following this, a number of studies found an increased mortality risk when using the cutoff lactate of >2.0 mmol/L (26, 27). This led to the gradual adoption of 2.0 mmol/L as the cutoff that defines the reference range in contemporary literature and in the latest national and international guidelines (1, 22). In our study, when we looked at the mortality among septic patients, relative hyperlactatemia patients had a higher mortality (4.4 vs. 1.9%). They were also more likely to receive more antibiotics, vasopressors, and IV fluids at 48 h. A study by Trzeciak et al. found that 70% of patients diagnosed with an infection had lactate levels below 2.00 mmol/L and a mortality rate of 15% (28). Recent evidence suggests that even relative hyperlactatemia in patients with septic shock is predictive of mortality (14, 16, 19, 29). Furthermore, Shetty et al. found that patients with lactate levels between 1.00 and <2.00 mmol/L had increased in-hospital mortality (OR 2.93) compared to patients with lactate levels <1.00 mmol/L (27). All of these studies are in line with our results, which show that relative hyperlactatemia patients had 1.78 times greater odds of in-hospital mortality. Despite all this evidence, there remains a knowledge gap surrounding relative hyperlactatemia in the ED, and it is still unknown whether patients with relative hyperlactatemia should be treated differently. Without a more nuanced understanding of lactate levels below the reference range of 2.00 mmol/L, ED physicians may be falsely reassured.

Similar to our results, Rishu et al. in the ICU specifically sought to determine the cutoff for lactate within the reference range that has the greatest prognostic value (21). Using the Youden index, they found that the optimal cutoff was 1.35 mmol/L and that relative hyperlactatemia above that cutoff was associated with increased hospital mortality (OR 1.60). Our study also shows that relative hyperlactatemia compares to APACHE II, a well-studied risk stratification score that correlates with mortality in critically ill patients (30). Indeed, relative hyperlactatemia patients had a higher APACHE II score than patients with lactate <1.33 mmol/L. This study adds stronger evidence that lactate within the normal range should be interpreted cautiously, as it may still be associated with an increased mortality.

## Limitations

To the best of our knowledge, this investigation is the first to explore relative hyperlactatemia in a population of adult ED patients. It is important to note that our results have shown that relative hyperlactatemia patients were older and had higher rates of comorbidities, laboratory derangements, vasopressor use, steroid use, in-hospital mortality, and IV fluid use. Our strengths include the large sample size and the standardized lactate measurement in the same laboratory for all patients. This study is limited by its retrospective nature and is thus prone to selection bias. As such, our results and our study should be viewed as an observation. These results need to be corroborated by a prospective trial in order to have any clinical impact. Second, our inclusion criteria were restricted to patients on whom there was a clinical decision to draw a serum lactate level, and this also might have introduced a selection bias. Third, since the data pertains to a single center, there may be limitations to the generalizability of the findings. In an attempt to minimize information bias, the authors held multiple meetings to ensure correct patient identification and underwent training to standardize the rigorous data abstraction protocol. The data collected is insufficient to define the most likely etiology of the relative hyperlactatemia or the effect of early interventions in patients with relative hyperlactatemia on clinical outcome, although this was beyond the scope of this study.

## Conclusion

The optimal cutoff of initial serum lactate within the reference range that discriminates between survivors and non-survivors in the ED is 1.33 mmol/L. Relative hyperlactatemia is associated with increased in-hospital mortality in patients presenting to the ED regardless of diagnosis or comorbidities. Further studies are required to determine the optimal management for patients with relative hyperlactatemia.

## Data Availability Statement

The raw data supporting the conclusions of this article will be made available by the authors, without undue reservation.

## Ethics Statement

This study was approved by the Hospital's Institutional Review Board (IRB) (BIO-2018-0453). Written informed consent for participation was not required for this study in accordance with the national legislation and the institutional requirements.

## Author Contributions

RB, GA, and HT made substantial contributions to the conception and design of the study. RB, SJ, RS, NM, KA, NK, HT, and MM were responsible for acquisition of data, analysis, and interpretation of data. RB, GA, and SJ were involved in drafting the manuscript. GA and RB were responsible for revising the manuscript critically for important intellectual content. RB takes responsibility for the paper as a whole. All authors contributed to the article and approved the submitted version.

## Conflict of Interest

The authors declare that the research was conducted in the absence of any commercial or financial relationships that could be construed as a potential conflict of interest.

## Abbreviations

ED: emergency department
ICU: intensive care unit
GPU: general practitioner unit
COPD: chronic obstructive pulmonary disease
CHF: congestive heart failure
SOFA: sepsis-related organ failure assessment
ICD: International Statistical Classification of Diseases
LOS: length of stay
SPSS: Statistical Package for Social Sciences
ESRD: end-stage renal disease
qSOFA: quick sepsis-related organ failure assessment
WBC: white blood cells
ANC: absolute neutrophil count
BUN: blood urea nitrogen
INR: international normalized ratio
PT: prothrombin time
PTT: partial thromboplastin time.
